# Comparative evaluation among laser-treated, machined, and sandblasted/acid-etched implant surfaces: an in vivo histologic analysis on sheep

**DOI:** 10.1186/s40729-019-0204-4

**Published:** 2020-02-19

**Authors:** I. De Tullio, M. Berardini, D. Di Iorio, F. Perfetti, G. Perfetti

**Affiliations:** 1 Department of Medical, Oral and Biotechnological Sciences, University of Chieti-Pescara, Chieti, Italy; 2 Pescara, Italy; 3 Foggia, Italy

**Keywords:** Implant surface, Osseointegration, Bone to implant contact, Dental implants

## Abstract

**Purpose:**

The aim of the present in vivo analysis was to evaluate the osseointegration process of titanium implants with three different surfaces (machined, sandblasted and acid-etched, and laser-treated) after 15 and 30 days of healing period.

**Materials and methods:**

Thirty-six implants with different surfaces were placed in the iliac crest of four Bergamasca sheep. The implant surfaces tested were sandblasted and acid-etched (group A), laser-treated (group B), and fully machined (group C). Two animals were sacrificed after 15 days while the other two after 30 days. Histological and histomorphometric analyses were performed.

**Results:**

After 30 days, the bone tissue layer onto implant groups A and B appeared almost continuous with small marrow spaces interruption, while on the machined surface (group C), larger spaces with marrow tissue alternated with the bony trabeculae onto the titanium surface. Implants in groups A and B showed significantly higher implant contact percentage (BIC%) value than group C (*P* < 0.05). Moreover, it was observed a BIC% increase in both groups A and B between 15 and 30 days while in the machined group (group C), the BIC% decreased.

**Conclusion:**

Results from the present in vivo analysis revealed that both sandblasted/acid-etched and laser-treated titanium implants, compared to the machined ones, have higher values of osseointegration in less healing time.

## Introduction

Dental implant surfaces represent one of the key factors that could influence the osseointegration processes [1]. Puleo et al. [2] confirmed that the surface topography, as well as the chemical nature and the implant macro and micro geometry, is involved in creating a clinical and histological efficient bone-implant interface. It was demonstrated that different superficial treatments could affect significantly both the amount of bone directly contacted to the titanium (bone to implant contact percentage) and the speed over the time of bone apposition onto implant surface [3, 4]_._

Implant surfaces are divided, on the microstructural point of view, into “smooth” (generally defined as “machined”) and “rough,” obtained by milling, sandblasting, and/or acid etching procedures [5]; normally, the distinction between smooth and rough surfaces is based on the measurement of surface roughness (Ra parameter) [6]. The bone amount onto the titanium surface is greater when using rough surfaces than smooth ones [7].

However, some authors theorized that bone-forming cells seem to be more influenced by the micromorphology of the surface than by its roughness [8]. Perrotti et al. [9] proposed the fractal analysis as surface analysis method and speculate that the best results, in terms of bone to implant contact percentage (BIC%), are obtained by using implants with uniform surface morphology instead of those with irregular surfaces characterized by peaks and troughs. These results were confirmed by other studies that showed that bone-forming cells seem to have a particular affinity for titanium surfaces with a regular and uniform roughness [10, 11].

Titanium implant treatments, which enhance bone apposition rate, inevitably create surfaces with irregular patterns, and some manufacturing contaminants could remain over the implant [12]. These materials could interfere with the new bone apposition process [13].

The laser treatment of titanium surfaces represents an innovative implant manufacturing technique that obtains a uniform and pure implant surface. This peculiar treatment uses high-density energy density by focalizing the laser source to melt, to heat, to sublimate, and to modify the superficial layers of the materials titanium by sublimation. Laser treatment allows setting the parameters that determine the roughness of the implant to obtain a micrometric porosity perfectly reproducible in shape, diameter, and depth. The laser surface treatment is also an effective method to obtain titanium surfaces free of contaminants because no acid or metal sand is needed during surface treatment processes [13]. Residual contaminants, which may remain onto the titanium surface after manufacturing procedures that involved acid or metals, could inhibit osseointegration [14].

In addition, some animal studies [15, 16] found an increased removal torque in laser processed implants compared to machined surface implants inserted.

The aim of the present paper was to evaluate the osseointegration process, in terms of bone to implant contact percentage (BIC%), of three different implants surface (machined, sandblasted and acid-etched, and laser-treated) both after 15 and 30 days of healing time.

## Materials and methods

The Ethics Committee for Animal Research of the Veterinary School of the University of Teramo (Teramo, Italy) approved the study protocol, which followed guidelines established by the European Union Council Directive of February 2013 (R.D.53/2013).

A total of 36 implants were used in the present study. Implants had different macro-geometries and surfaces and they were divided into three groups of 12 implants each.

**Fig. 1:**
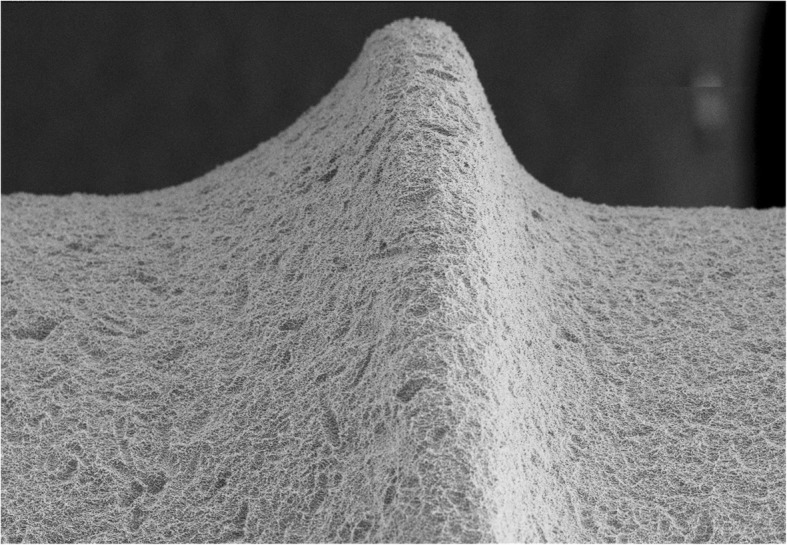
Scanning electron microscopy picture of group A implant surface

Twelve implants, belonged to group A, were 4.1 mm in diameter and 10 mm in length, with a smooth neck of 2.8 mm and SLActive sandblasted and acid-etched surface (STRAUMANN Basel, Switzerland) (Fig. 1).

**Fig. 2:**
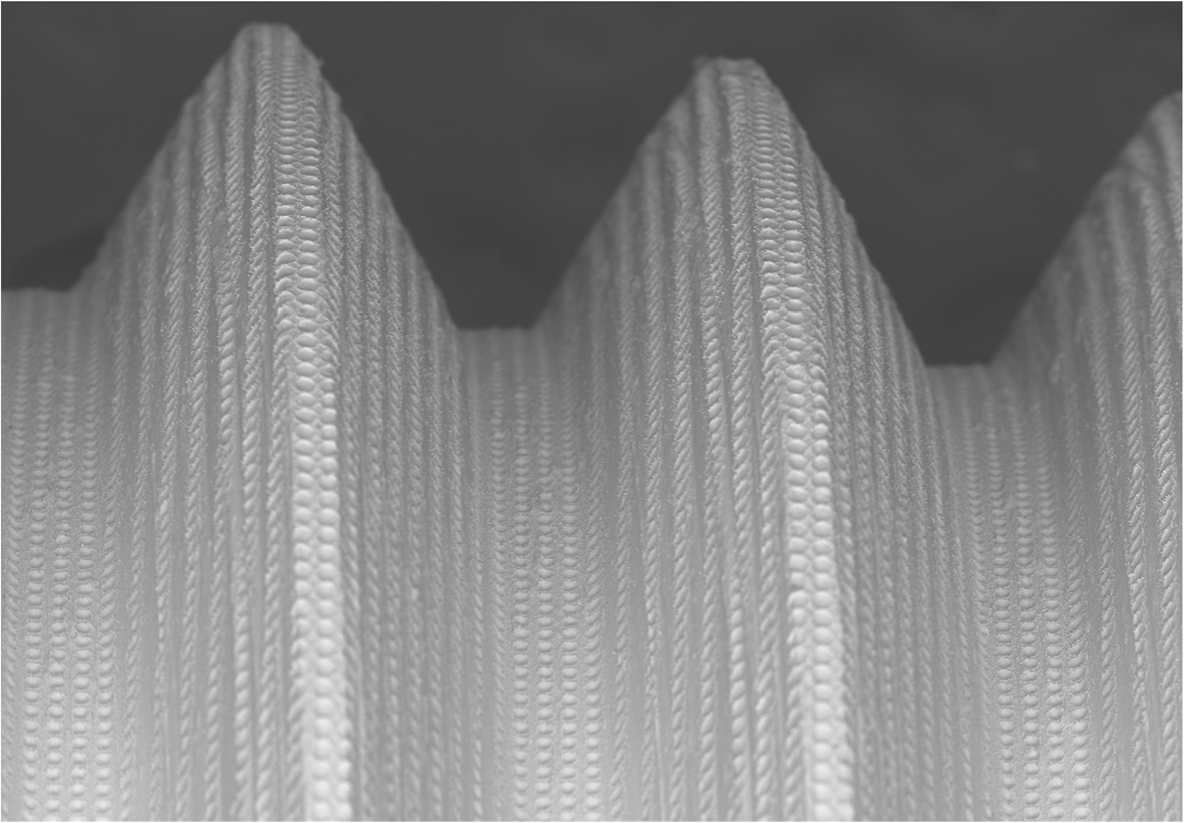
Scanning electron microscopy picture of group B implant surface

Twelve implants, belonged to group B, were 3.8 mm in diameter and 11 mm in length and showed a small thread design, a smooth neck of 0.25 mm in the most coronal area, a micro-threads collar of 3.25 mm in length and laser-treated surface (GEASS s.r.l. Pozzuolo Del Friuli, Italy) (Fig. 2).

**Fig. 3:**
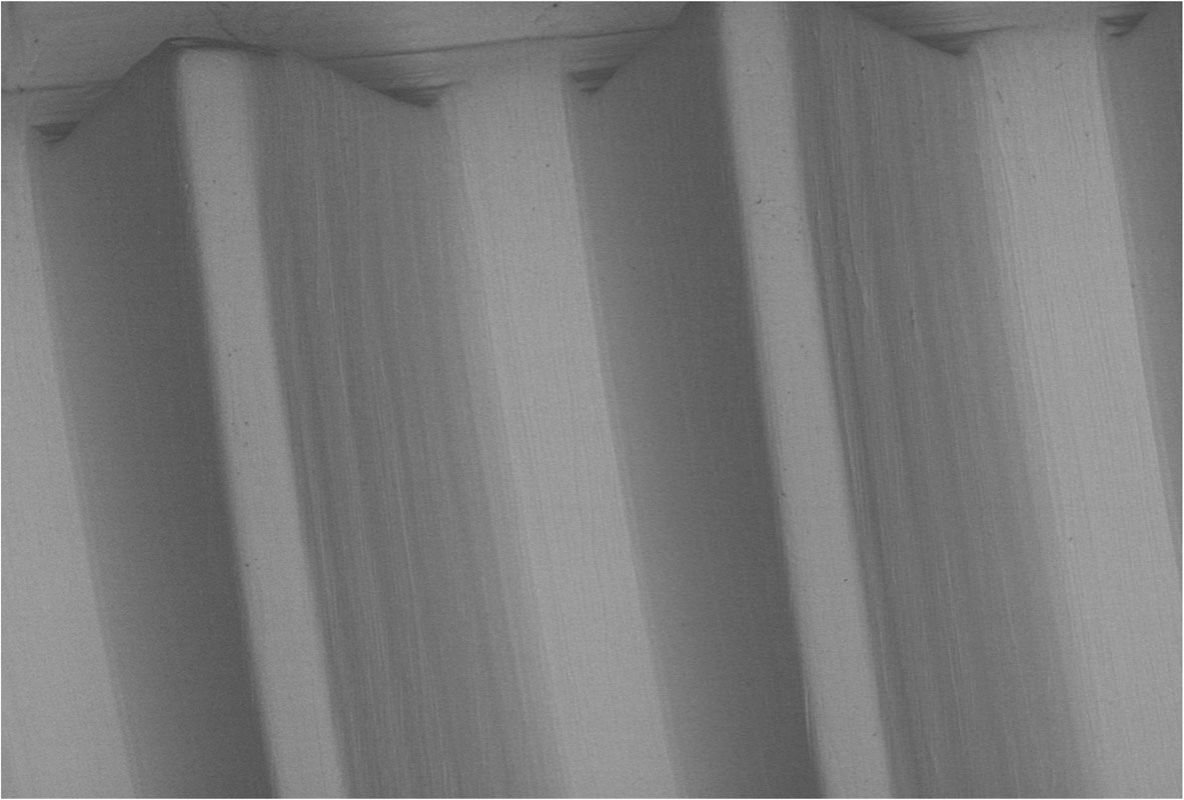
Scanning electron microscopy picture of group C implant surface

Twelve implants, belonged to group C, were 3.8 mm in diameter and 11 mm in length and showed a small thread design, a smooth neck of 0.25 mm in the most coronal area, a micro-threads collar of 3.25 mm in length and machined surface (GEASS s.r.l. Pozzuolo Del Friuli, Italy) (Fig. 3).

Implant details of both groups are shown in Table 1.

**Table 1:** Implants details of both groups: screw pitch, smooth neck length, surface treatments, and roughness

	Group A implants	Group B implants	Group C implants
Screw pitch	1.25 mm	0.6 mm	0.6 mm
Smooth neck	2.8 mm	0.25 mm	0.25 mm
Surface treatment	Large grit-blasted and acid-etched SLA surface, processed to a high degree of hydrophilicity (SLActive®)	Laser surface characterized by a series of 20 μm diameter holes (7–10 μm deep) every 10 μm (Syntegra®)	Machined surface
Surface roughness (Ra)	1.5 μm	0.37 μm*	0,75 μm

*Value obtained considering the holes not as part of the roughness but as part of the primary profile. R_a_ inside the holes is 0.1 μm while outside the holes is 0.4 μm

Four female Bergamasca sheep, 4–5 years old, were included in the study. Clinical examination determined that all animals were in good general health. Exclusion criteria included general contraindications (pregnancy, systemic disease) to implant surgery and active infection or severe inflammation in the area intended for implant placement.

All animals underwent deep sedation with xylazine hydrochloride 0.1 mg/kg intravenously and 0.2 mg/kg intramuscularly (Bayer–Leverkusen, Germany). After administration of deep sedation, trichotomy was performed, as well as cleaning and disinfection of surgical sites through by using soaped povidone-iodine 7.5%; loco-regional infiltrative anesthesia of lidocaine hydrochloride solution at 2% followed (Bioindustria, Novi Ligure, Italy).

**Fig. 4:**
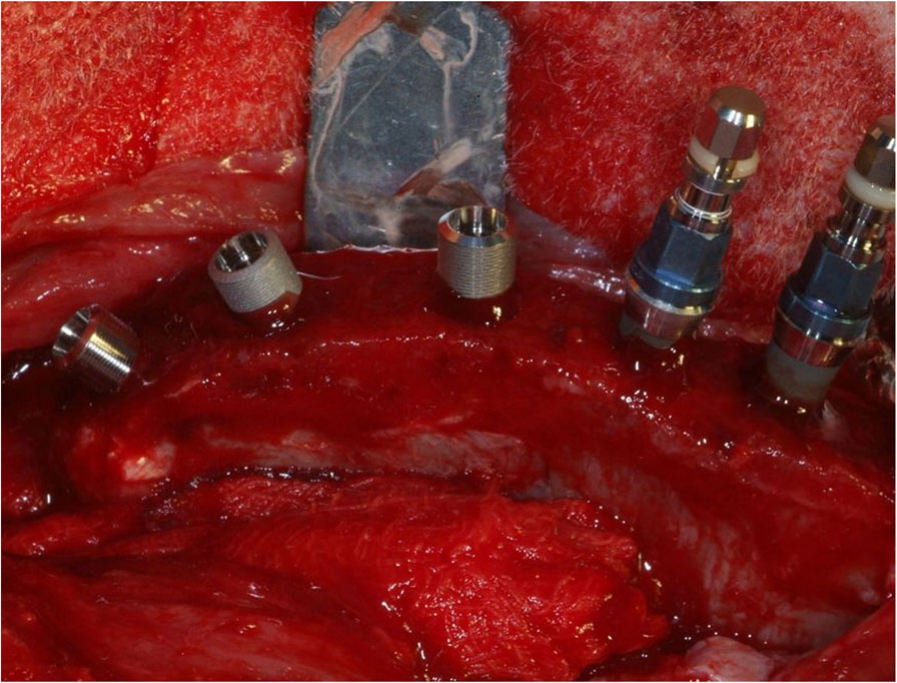
Exemplificative photo of implant placement in sheep iliac crest (left side). All implant groups were inserted in the same bone host

The edges of iliac crests were exposed through a skin incision of 15 cm in length. The skin and facial layers were opened and closed separately. After dissection of the soft tissues, the bone was exposed and five osteotomic sites were prepared in the left side and four in the right one of the iliac crest for a total of nine osteotomic implant sites in each animal (Fig. 4).

Implant drilling procedures were carried out using the drill sequence recommended by the manufacturer. The drill speed was set at 700 rpm under continuous sterile saline solution irrigation (stored at + 4 °C).

Implants were inserted with an insertion torque peak between 28 and 34 Ncm. Each animal received three implants of each group.

The suture of deep muscle planes was performed with polyglycolic acid Dexon II (Kendall Company, MA, USA), while the superficial soft tissues were sutured with a non-absorbable suture (Codisan S.p.A., Belpasso, Italy).

The surgical site underwent to topical antibiotic therapy (Gellini-Intervet, Milan, Italy). Finally, each animal was subjected to systemic antibiotic postoperative therapy with 20 mg/kg of intravenous ampicillin every 12 h for 3 days after surgery.

Two animals were sacrificed by intravenous injection of Tanax (Intervet - Boxmeer, Netherlands) after 15 days, while the other two animals after 30 days.

All bone specimens were immediately rinsed in saline solution, fixed in 10% neutral buffered formalin and finally processed to obtain thin ground sections.

Afterwards, the samples were included in resin (LR White EM, TAAB Laboratories Equipment Ltd., England) and sectioned along the longitudinal plane with a microtome (Micromet, Bologna, Italy). From each sample, approximately four sections were obtained with a 300 μm thickness; the slides were then reduced in thickness to about 90 μm, using a lapping machine (Micromet, Bologna, Italy). Subsequently, the sections were stained with toluidine blue and magenta acid and analyzed under an optical microscopy (Laborlux Leitz, Leica Microsystems, Wetzlar, Germany) equipped with a digital camera (3CCD JVC KY-F55B, JVC, Yokohama, Japan).

The resulting images have undergone a histological qualitative and quantitative morphometric analysis by means of dedicated software (Image J 1.32j. Wayne Rasband, National Institutes of Health, USA) to calculate the BIC% values. On each image (× 9 magnification), a midline parallel to the long axis of the fixture was traced using a software for graphic processing (Corel Photo Paint, Corel Corporation, USA) to divide the image into two halves. The two different slices were then treated separately during the intermediate steps of BIC% measurement, according to the following order:

A. Measurement of the total length of the left half of the fixture;
B. Measurement of the contact area between bone and implant in the left half of the fixture;
C. Measurement of the total length of the right half of the fixture; and
D. Measurement of the contact area between bone and implant in the right half of the fixture.

Afterwards, the sum of parameters A and C represented the total length of the whole implant (wIMP) while the sum of parameters B and D the total bone in direct contact (tBIC) with the implant surface. A value was obtained by the ratio between tBIC and wIMP which, compared to the unity, provided the percentage of BIC calculated for the single image (BIC%). The obtained BIC values from each group of samples were finally submitted to descriptive statistics and inferential analysis by using a specific software (SYSTAT 9.0; SPSS Science Software GmbH, Erkrarth, Germany).

Ordinary one-way ANOVA test was applied to test the statistical differences between the mean BIC% values of the groups using the statistical software GraphPad Prism 8.3.0 (www.graphpad.com).

*T* test was used to compare the BIC% mean value between 15 and 30 days of each group

## Results

All implants resulted clinically integrated and stable into the bone tissue. No signs of tissue inflammation or infection were detected.

### Histological qualitative analysis

#### Samples collected after 15 days of healing

**Fig. 5:**
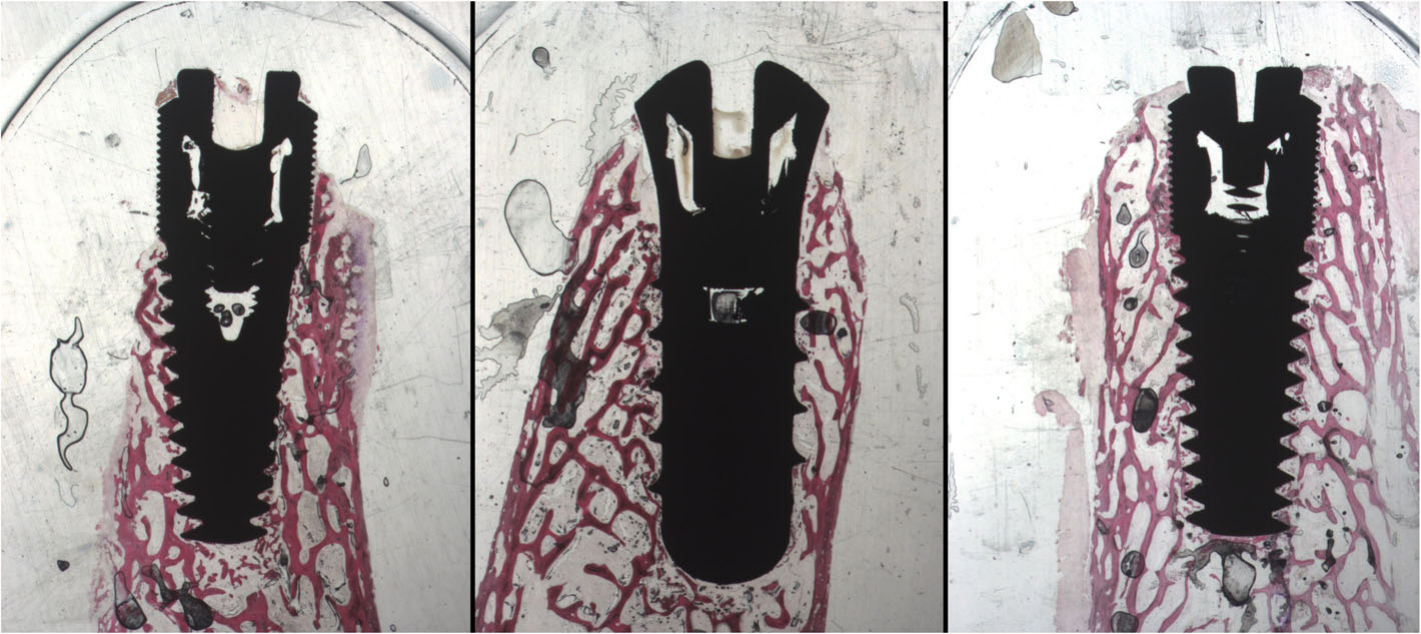
Optic microscope photo (× 9 magnification) after 15 days of implantation. Left side: machined implant (group C). Central photo: sandblasted and acid-etched implant (group A). Right side: laser-treated implant

At low magnification, all the samples appeared surrounded by new tissue. The distinction between native tissue and newly formed bone was not clear, likely due to the fact that the latter is still in an initial forming phase. In the machined samples (group C) bone fractured trabeculae were present around the fixture apex (Fig. 5).

In some samples, belonging to the groups A and B, however, it is possible to observe some areas where a thin osteoid matrix band was directly contacted to the implant surface.

#### Samples collected after 30 days of healing

**Fig. 6:**
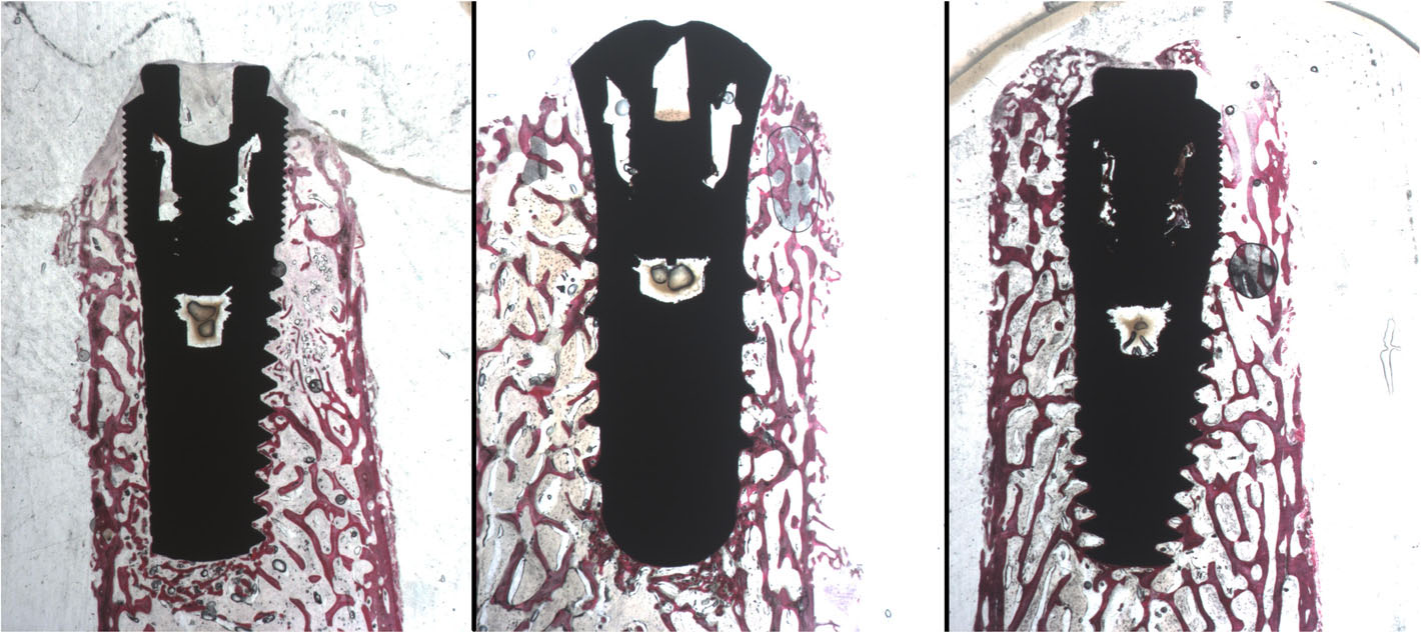
Optic microscope photo (× 9 magnification) after 30 days of implantation. Left side: machined implant (group C). Central photo: sandblasted and acid-etched implant (group A). Right side: laser-treated implant

All the samples appeared surrounded by bone tissue. A thin layer of newly formed bone covered implant threads. Newly formed bone connected the fractured bone trabeculae to bone fragments and/or to the implant surface. Implants belonging to groups A and B were observed more osteogenesis areas and mineralization nuclei than implants of group C (Fig. 6).

The bone tissue layer onto SLActive and laser-treated surfaces appeared almost continuous with small marrow spaces interruption, while on the machine surface larger spaces with marrow tissue alternated with the bony trabeculae onto the titanium surface.

### Histomorphometric quantitative analysis

No implant failure was detected during the follow-up period. After 15 healing days, the mean BIC% was almost the same in groups A and B while machined implants (group C) revealed the lowest osseointegration rate value. BIC% differences between the groups were statistically significant (*P* < 0.05). All BIC% mean values of each group were displayed in Table 2.

**Table 2:** Mean BIC% value of each group after 15 days of healing

Group	BIC% (mean ± SD)
A	39.08 ± 15.85
B	37.35 ± 15.76
C	25.28 ± 8.97

After 30 healing days, groups A and B showed better osseointegration values compared to those at 15 days. Group B implants showed BIC% value significantly higher (*P* > 0.05) in respect with those at 15 days. Group C showed a mean BIC% value lower than that observed at 15 days. BIC% differences between groups were significant (*P* < 0.05). All BIC% data, after 30 days, were summarized in Table 3.

**Table 3:** Mean BIC% value of each group after 30 days of healing

Group	BIC% (mean ± SD)
A	50.31 ± 13.44
B	56.53 ± 13.62
C	20.54 ± 11.06

## Discussion

In the present study, the iliac crest of the sheep was chosen as a model because the site is characterized by a cancellous bone rich in marrow spaces, similar for quality to D4 density. This bone model appears superimposable to postero-lateral sectors of the human upper jaw that often represents a hard challenge for implant osseointegration due to low bone density.

Bone quality, in fact, is a key factor in dental implant rehabilitations because it could significantly influence the bone percentage of implant osseointegration [17]. In D4 bone type, it could be difficult to achieve a sufficient primary and secondary implant stability, and it is important to achieve high and fast, as much as possible, bone apposition onto titanium surface. The present analysis focused its attention on the bone affinity of different implant surfaces, evaluating the speed of bone formation and the amount of newly formed tissue.

For this reason, the sampling procedure was performed in short term (15 and 30 days) to be able to better investigate the differences on bone apposition speed onto different titanium implant surfaces.

The speed of bone apposition onto titanium surface could improve implant stability during the crucial initial healing phase allowing immediate or early loading protocol. It is important to underline that the new bone formation process, onto the implant surface, represents the transition between the initial primary implant mechanical stability to the functional secondary implant stability [18]. Once the osseointegration and the subsequent secondary stability are achieved, the implant may undergo to the occlusal loads with success [19].

The BIC%, observed by the present analysis, after 15 and 30 days of the machined surface group was very low if compared to the other groups. This fact strongly suggests the existence of more rapid integration phenomena in sandblasted and acid-etched surfaces and in laser-treated ones.

Moreover, it was observed a BIC% increase in both groups A and B between 15 and 30 days while in the machined group (group C), the BIC% decreased. This is because a slow physiological process of reabsorption of the fractured bone trabeculae (due to implant drilling procedures) characterizes the bone tissue in contact with any surface, but in group C, the newly formed bone apposition seems to be subsequent. In groups A and B instead, the micro-geometry of the surface could enhance the adhesion and proliferation of the bone-forming cells, and the new bone formation phase seems to start from the first days concurrently with the remodeling of the old bone trabeculae.

Data showed by the present study suggest that laser and sandblasted and acid-etched surface treatments could enhance the osteogenic bone formation by “contact,” already observed by other authors [20, 21].

Another interesting emerging datum is the observation of BIC% changes between 15 and 30 days: it is possible to assume that between the fifteenth and the thirtieth day, most part of the peri-implant bone apposition occurs in laser and sandblasted and acid-etched surfaces.

A previously published study [22], comparing machined to laser-treated surfaces, found clear and significant differences between the two surfaces in the amount of bone and significantly better secondary stability (measured by removal torque values).

Results of the present paper were confirmed by a study that observed good osseointegration and no significant differences in the BIC% at 2 or 4 weeks comparing sandblasted/acid-etched and laser-treated surfaces. They also found that the laser-treated surface was cleaner and more uniform than the SLA surface [23].

The smooth surface implants, used in the present in vivo evaluation, appeared integrated in the host bone with low values of bone to implant contact percentage in respect with rough surfaces, and this datum is well documented in the international literature [24, 25].

Other authors [26] also found no significant differences both in biomechanical strength and in implant stability between laser-etched and SLactive implant surfaces.

Sinjari et al. [27] also evaluated the effects of different titanium surface treatments on blood clot formation, and they demonstrated in vitro that the laser-conditioned surface, although it has a low roughness value (Ra of 0.25  ±  0.02 μm) compared to a standard grit-blasted surface (Ra of 1.30  ±  0.03 μm), had higher wettability and blood clot extension in respect with machined and rough surfaces.

Some authors [28] evaluated, in vitro, the biofilm formation of *Porphyromonas gingivalis* on titanium disks with different surface topographies. They analyzed a total of 96 disk-shaped specimens of laser-treated, sandblasted, and machined surfaces and they found that titanium grade 4 with laser topography appears to be significantly efficient in the reduction of the *P. gingivalis* biofilm formation. Data from this study demonstrated that the laser-treated implant surface allows osseointegration percentages entirely comparable to sandblasted and acid-etched surfaces. The innovation of this new laser surface treatment would seem to be in the fact that the poor roughness is more difficult to colonize from the bacteria responsible for peri-implant diseases.

## Conclusions

Results from the present in vivo analysis revealed that both sandblasted and acid-etched titanium implants and laser-treated titanium implants, compared to the machined ones, have higher values of osseointegration in less healing time.

Indeed, both groups A and B at 15 days had higher values of the BIC% if compared to group C and were able to significantly increase their BIC% in the passage from 15 to 30 days, allowing safe early occlusal load protocol.

The superior initial bone feeling could be ascribed to the micro-geometry that ensures great osteoblasts adhesion and proliferation.

Data of the present study confirmed that the use of laser or sandblasted and acid-etched implant surfaces should be preferable to machined ones, especially in the case of low-density bone or immediate loading protocols.

Future clinical studies are needed to confirm the results of the present in vivo animal study.

## Data Availability

All data and materials are available at University Chieti-Pescara, Department of Medical, Oral and Biotechnological Sciences, University of Chieti-Pescara, Chieti, Italy.
